# Restaurant Diners’ Switching Behavior During the COVID-19 Pandemic: Protection Motivation Theory

**DOI:** 10.3389/fpsyg.2022.833627

**Published:** 2022-05-27

**Authors:** Hamid Mahmood, Asad Ur Rehman, Irfan Sabir, Abdul Rauf, Asyraf Afthanorhan, Ayesha Nawal

**Affiliations:** ^1^Faculty of Business and Management, Sultan Zainal Abidin University, Kuala Terengganu, Malaysia; ^2^Faculty of Business and Management, University of Central Punjab, Lahore, Pakistan

**Keywords:** vulnerability, regret, altruistic fear, brand awareness, switching intention, switching behavior

## Abstract

The unsettling fear of COVID-19 infections has caused a new trend in consumer behavior in the food and beverage industry. The unprecedented COVID-19 pandemic has shifted consumers’ preferences from eat-in to online delivery. This research aims to measure the impact of consumers’ motivation to protect themselves from contracting COVID-19, which explains why people switch from eat-in to online food delivery. We adopted the theory of protection motivation (PMT) to explain consumer switching behavior during the COVID-19 pandemic. The present study investigated the mediating effect of switching intention on the relationship between vulnerability, altruistic fear, anticipated regret, and switching behavior. Simultaneously, we examined the role of brand awareness as a moderator of behavioral choices of consumers switching from eat-in to online delivery. We collected data from 681 eatery consumers in Kuala Terengganu, Malaysia, using scenario-based survey questionnaires (327 eat-in respondents and 354 online delivery respondents). Then, the data were analyzed using structural equation modeling (SEM). This new generation analysis was conducted using the analysis of moment structure (AMOS) (v.24.0) and the statistical package for social science (SPSS—version 25.0). The results indicated that consumer vulnerability, altruistic fear, and anticipated regret of COVID-19 increased consumers’ propensity to shift from eat-in to online food delivery. Allegedly, consumer behavioral control and intention of switching toward online delivery were pointedly affected by switching behavior. The results indicated that consumer vulnerability, altruistic fear, and anticipated regret of COVID-19 increased the shifting of restaurant dine-in patterns and made the intention to switch to online delivery. Consumers’ alleged behavioral control and their intention of switching toward online delivery were pointedly affected by switching behavior. We also found that brand awareness moderately affects switching behavior toward restaurant settings. The present research contributes to developing the consumer behavior model of switching from eat-in to online delivery. This study also provides eatery customers and the business community with a safer and healthier proposition of shifting to online food delivery during the pandemic.

## Introduction

In December 2019, when the first reported case of the coronavirus disease in China, which is also famously known as COVID-19, 77.8 million reported cases of COVID-19 have been confirmed. Furthermore, more than 4.3 million deaths were reported worldwide on December 11, 2021 (Worldometer, 2021). The worldwide spread of COVID-19 has caused local governments to impose movement restriction orders or lockdowns to mitigate the spread of the virus. Previous research in Malaysia (Marzo et al., 2021), China (Qiu et al., 2020), India (Varshney et al., 2020), Italy (Mazza et al., 2020), the United States, and Canada (Taylor et al., 2020), and Germany (Petzold et al., 2020) found that a large proportion of their populations shows signs of despair, post-traumatic stress disorder, and anxiety. Even worse, the COVID-19 pandemic has forcefully changed our daily routines. Countermeasures, such as quarantine, limited business operation hours, working from home, and border closures, have altered the job market, consumer behavior, and government policies. A pandemic during a modern period has posed a severe threat to humanity, especially mental wellbeing and mobility. The current studies of COVID-19 in the context of mental wellbeing indicate that numerous people are reported to have fear, stress, and anxiety-related symptoms (Casagrande et al., 2020; Wang et al., 2020) and perceived vulnerability resulting from the transmittable disease outbreak (Bakioglu et al., 2020; Jungmann and Witthoft, 2020). Recent extant studies on Ebola and SARAS pandemics also revealed the outbreak of transmittable diseases (Boyraz and Legros, 2020; Taylor et al., 2020).

According to Global Data Analysis [GDA] (2020), the countermeasures taken by the authorities to mitigate the spread of COVID-19 have a significant impact on the macro and micro-economy. The OECD (2020) stated that the global economy could shrink by somewhere between 2.4 and 2.9%, and the unemployment rate is projected to surpass the level of the 2008 crisis. Compared with other sectors, the COVID-19 pandemic had a devastating impact on the food and beverage industry because of the withheld spending due to the lockdowns and movement restriction orders (Gossling et al., 2020). Food and beverage businesses are suffering as a result of the pandemic. Statistically, about 4 out of 10 restaurants had to close at the commencement of the COVID-19 surge (National Restaurant Association [NRA], 2020). Restaurant sales have reached the lowest level over the past 35 years, and their loss was predicted at almost $240 billion (National Restaurant Association [NRA], 2020). The restaurant industry is highly acknowledged for its vulnerability to pandemics, particularly the fear of contracting COVID-19 due to the poor social distancing at its eatery premises (Gossling et al., 2020).

Interestingly, this pandemic altered consumer behavior and perception of the dine-out culture. Typical diners are now reluctant to eat in restaurants due to the fear of contracting COVID-19 disease, resulting in being hospitalized or, even worse, death. This circumstance has deviated consumer intention from eat-in to online food delivery. This shift in intention is referred to as “restaurant at home,” meaning that consumers engage in-home delivery services rather than going to a restaurant to dine (Sheth, 2020). During the lockdowns, consumers who had never experienced online food delivery changed their preferences for this online service (Watanabe and Omori, 2020). This change in consumers’ intention toward online food delivery is affected by an individual’s anticipated regret of making a wrong decision on selecting between an online food delivery service and an eat-in restaurant (Connolly and Butler, 2006; Zeelenberg and Pieters, 2007). The anticipated regret (consumers’ expected regret when they decide to switch from eat-in to home delivery) affects the consumer’s switching intention. Consumers who switch to online food delivery will compare the new experience with the dine-in experience, resulting in regret (Liao et al., 2017). Considering the negative and positive effects that anticipated regret has on consumer switching intentions, there are contradictory findings in this context. Furthermore, this contradiction created a gap in the existing literature.

To bridge this gap in the literature, the current study analyzes the impact of vulnerability, altruistic fear, and anticipated regret on switching intention, consumer switching behavior, and brand awareness to further investigate the role of switching behavior to switching intention. Additionally, the present research contributes to the literature in three ways. First, this research was conducted amid the COVID-19 pandemic. This study invokes natural experiments and provides a timely investigation into consumer behavior regarding dine-in and home delivery in real-time crises. Secondly, the current study proposes and tests a research model to demonstrate switching intention and switching behavior in the COVID-19 pandemic. Thirdly, to our best knowledge, this research was first conducted in a restaurant context in Pakistan to switch from dine-in to online food delivery during the COVID-19 pandemic.

Furthermore, at the commencement of this research, different countries experienced different stages of the COVID-19 pandemic. China seems to have COVID-19 under control from where it started, but other countries in the reign, e.g., Pakistan, Malaysia, India, and Indonesia, are approaching their peak in the COVID-19 surge. This unique situation provides valuable insight into how consumer switching behaviors change with different stages of pandemic engagement in switching intention, which has practical implications for policymakers and restaurant businesses in providing a more robust response to the global crisis.

## Literature Review

### Roger’s Theory of Protection Motivation

Rogers (1975) introduced the protection motivation theory, which explains individual protective behavior regarding threat stimulus. Under PMT theory, Roger introduced two elements, namely, coping appraisal and a distinct cognitive process (the threat appraisal) (Rogers, 1983). Furthermore, the cognitive process refers to threat appraisal, which is used to evaluate threat levels by individuals. Threat appraisal contains important antecedents of adaptive actions by individuals: threat severity and threat vulnerability (Rippetoe and Rogers, 1987). According to PMT, the perception of vulnerability initiates individuals to become involved in risk-preventive behavior (Rippetoe and Rogers, 1987). Numerous studies tested protection motivation variables in 2009 during the influenza pandemic to measure behavioral intention in engaging in self-protective behavior (Prati et al., 2012; Cho and Lee, 2015). Nevertheless, few studies applied protective motivation variables to measure consumer behavior in the current COVID-19 pandemic (Kim et al., 2021).

Although PMT was well validated in previous pandemic studies, the unprecedent condition of COVID-19 brings the restaurant industry to a critical examination of PMT theory in a new pandemic context (Kim et al., 2021). It was also seen that the impact of the previous pandemic was more confined than the current pandemic, which was noticed without exception and with prolonged effect (Sharma et al., 2021). Due to restrictions on travel, social distancing, and lockdown, the restaurant industry is facing a massive decline in customer traffic and profits. According to the National Restaurant Association [NRA] (2020), the restaurant industry is still not recovering, which indicates lower customer traffic and sales. Therefore, it is time to study PMT constructs regarding changing consumption patterns and emotional responses during COVID-19 outbreaks.

### Perceived Vulnerability and Switching Intention

Perceived vulnerability is an imperative factor in the threat appraisal process, suggesting that the greater the perceived threat, a person performs more precautionary behavior (Rogers, 1983). Generally, individuals underestimate their vulnerability to illness or other infectious diseases (Weinstein, 1989). However, the rapid spread of life-threatening COVID-19, combined with the ongoing denial of these pandemic-related gratifications *via* mass media and various etymologies, creates a pandemic sense of vulnerability (Boyraz et al., 2020). By motivating individuals, understanding who is vulnerable to pandemics aids vital function in engaging in self-protective behavior (Bavel et al., 2020). However, the vulnerability during the COVID-19 pandemic undermines the sense of control and safety, increasing insecurity, loss of control, and disease-related worries (Boyraz et al., 2020), which trigger the intention to switch from dine-in to online food delivery. In the current COVID-19 pandemic, customers’ concerns can increase regarding dining in a restaurant and can be sensitive to the services they obtain (Min et al., 2021).

Additionally, behavioral responses to pandemic infection threats may be associated with perceptions of infection and personal risk, along with discomfort at restaurants associated with the risk of infection (Stangier et al., 2021). Past studies advocate that perceived vulnerability influences customer perception of service providers (Min et al., 2021). Furthermore, people prefer to stay at home when there is a surge in illness and infection risk (Teasdale et al., 2012). Similarly, when people perceive COVID-19 infection as a threat while dining at a restaurant, they tend to switch to online food delivery to avoid physical interaction.

***Hypothesis 1:** Perceived vulnerability is positively related to switching intentions among restaurant consumers.*

### Altruistic Fear and Switching Intention

Fear is a natural response that triggers epidemics and disease outbreaks to keep people from risky behavior and danger (Harper et al., 2020). Fear can also threaten self-interest and initiate a fighting response (Barbalet, 2001). Although PMT describes the cognitive appraisal process using coping and threat variables, these progressions contain personal fear assessment. Concerns related to the COVID-19 scenario when corona disease spreads rapidly with a high mortality rate and fears related to the assessment should be considered personal ones (Youn et al., 2021). During the COVID-19 pandemic, fear of getting infected is a foremost concern and an imperative construct in quantitative research, and altruistic-related fear refers to fretting about community members, households, healthcare workers, and others (Shim et al., 2012). Previous studies suggest that higher COVID-19 fear is associated with health compliance and prevents behavior such as social distancing (Yıldırım et al., 2021). Alfaro et al. (2020) argue that communities and individuals with a high level of altruism during the COVID-19 pandemic showed a low degree of mobility due to fear and threat from disease. Furthermore, COVID-19 outbreaks make people more threatened as altruistic fear increases, and, essentially, people fear that their family members or friends will die with the coronavirus (Sloan et al., 2020). Additionally, people believe in staying at home (Youn et al., 2021), which eventually switches their intention from dining in toward online delivery. Thus, COVID-19 increases the perception of fear among consumer behavior control or the ability to change behavior. Therefore, we argue that, when the consumer feels fear from COVID-19, this will increase the switching intention of consumers from dining into online food delivery.

***Hypothesis 2:** Altruistic fear is positively related to switching intention among restaurant consumers.*

### Anticipated Regret and Switching Intention

Anticipated regret refers to the negative nostalgic emotions people experience when they imagine or realize that the current situation could be different if they acted differently (Hatak and Snellman, 2017). However, numerous studies define anticipated regret as pre-behaviorally (Simonson, 1992; McConnell et al., 2000). According to Zeelenberg and Pieters (2007), people anticipate regret while switching because they feel they could make bad decisions, and anticipated regret affects their choice (Connolly and Butler, 2006). Although anticipated regret is based on comparisons of emotions, when people understand that the present situation is uncertain and an important decision, they will anticipate regret in the future (Zeelenberg and Pieters, 2007). Nonetheless, previous research has found that external risk increases consumer switching behavior (Youn et al., 2021). Although anticipated regret has not been studied as the leading factor of switching in the unique situation of the current COVID-19 pandemic, assessment of disease risk (vulnerability from COVID-19) can consider the external risk that encourages consumers to switch channels.

***Hypothesis 3:** Anticipated regret is positively related to switching intention among restaurant consumers.*

### Switching Intention and Switching Behavior

Consumer switching behavior is conceptualized as an economic phenomenon where consumers cease patronizing a particular supplier (Stewart, 1998). While switching intention is defined as transferring an existing supplier to a competitor (Bolton et al., 2004), in the COVID-19 context, consumer fear is a fundamental factor that might produce anxiety and stress (Bitan et al., 2020), impacting consumer buying patterns (Afthanorhan et al., 2019; Martinelli et al., 2021) and leading to switching behavior (Sheth, 2020). Additionally, several epidemic outbreaks have been seen in recent history, for example, SARS, Ebola, Swine Flu, dengue fever, and MERS (Balinska and Rizzo, 2009). The most predominant is that such outbreaks impact mitigation of health risk behavior (La Torre et al., 2009) and consumer behavior (Miri et al., 2020). During the pandemic, perceived severity, social norms, health knowledge, and response beliefs of individuals predict the adoption of preventive measures (Gamma et al., 2019), which have a considerable impact on consumer switching behavior (Latoo et al., 2020). Protection motivation theory also considerably explains human actions and motives during pandemics (Latoo et al., 2020). Our PMT study draws intention to the impact of individual threat and coping appraisal on consumer behavioral intention in COVID-19 outbreaks, which calls for research on factor-infusing consumer switching behavior. Relying on the above arguments, we hypothesized that consumers are shifting their intention from dining in toward online delivery due to the vulnerability of the disease, which has developed consumer switching behavior.

***Hypothesis 4:** Switching intention is positively related to switching behavior among restaurant consumers.*

### Switching Intention Mediation

According to Arora and Sahney (2018), existing research shows that consumers favorably evaluate coping actions when they perceive vulnerability, altruistic fear, and anticipated regret to the threat. For instance, consumers evaluate dine-in patterns negatively and shift their attitude toward online delivery when they observe a lofty ultimatum of psychological fear because of COVID-19. Additionally, in the current pandemic scenario, individuals prefer the adaptive actions of staying at home to prevent themselves and their families from infection. Based on the context, the switching intention of consumers’ restaurant patterns will sturdily affect their switching behavior (Hidayat et al., 2021). The present study categorizes the impact of protection motivation on consumers’ switching behaviors, ultimately describing their switching patterns.

Past studies examined whether altruistic fear (well-being of others) enlightened the switching intention during COVID-19 that triggered the switching behavior of consumers (Youn et al., 2021). Individuals in COVID-19 were found to be more vulnerable as their fear of altruism increased, encouraging them to switch their intention toward protective behavior such as social distancing (Sloan et al., 2020). In the pandemic situation of COVID-19, the evaluation of perceived vulnerability and anticipated regret can be precise as an external factor that encourages the consumer’s switching intention to switch behavior. Based on the extant studies, anticipated regret played a negative role in switching intention, but, in this study, COVID-19 positively contributed to switching intention. This way, we offer a unique direction to switch the behavior of consumers’ restaurant patterns by postulating a mediating role of switching intention.

***Hypothesis 5:** Switching intention mediates the relationship between perceived vulnerability, altruistic fear, anticipated regret*, *and switching behavior among restaurant consumers.*

### Brand Awareness as a Moderator

According to Tran et al. (2020), awareness plays a remedial role in consumers’ buying decisions, specifically for health-related products. Consumers were more conscious about their active lifestyles in the present era, which caused them to exaggerate their daily routine (Choi and Zhao, 2014). Furthermore, the consumers avoid frequent eating if they are concerned with nutrition (Bhuyan, 2011). Nutritionally, aware consumers will select the explicit ingredients sensibly and avoid the unwanted (Choi and Zhao, 2014). Brand awareness regarding health is the ultimate motive of consumers to buy food because it is an ecological learning and compelling health effort of buyers (Suki, 2013). Extant studies indicate that numerous consumers eat nutritious food for the sake of a healthy life (Jones, 2010; Suki, 2013). Such healthy practices behavior notifies the consumers’ requirements and inspirations regarding health (Baum et al., 1997). The elements of consumers’ actual behavior paved the way for behavioral intention because intention refers to the ability to engage in their behavior and actions. So, it is assumed that consumers have a full intention to switch toward hearty food and services when considering switching behavior. Such inferences do not acknowledge consumers’ behavior intentionally and specifically when influential factors are beyond their control (Ajzen, 2005).

Thus, we conjecture that the effect of brand awareness genesis is the intention of behavior to not convert to actual behavior. According to Brewer and Zhao (2010), the most prominent aspect of brand awareness is identifying the brand clearly, regardless of whether consumers have ever seen or heard about it. As a result, it can be assumed that brand awareness plays a significant role in consumer brand decisions. There are many marketing terms of communication, like social media, where consumers gain awareness regarding a brand. According to Shabbir et al. (2017), brand awareness is vital to liaise with the product consumers want. So, in the present era, it is the need of the hour to identify the consumers’ brand awareness extent regarding switching patterns of food services. Therefore, the association between the intention of switching and switching behavior might be moderated *via* brand awareness.

***Hypothesis 6:** Brand awareness influences the relationship between switching intention and behavior, so the relationship is weaker when brand awareness is high compared to low.*

## Methodology

### Context of the Study

Malaysia is chosen as the research setting because it’s hotel restaurants and institutional industries are distinct, fast-growing segments of the economy driven mainly through active tourism. According to The Straits Times (2021), with over 20,000 new cases of COVID-19 reported daily, restaurant patrons took precautions by choosing uncrowded places or those with outdoor areas. According to Ravindran (2021), if dine-in is prohibited for the next 1 or 2 months, about 60% of restaurant proprietors in the country will be forced to close permanently. A conservative approximation that 30% of the 200,000 food establishments was closed prior to the start of pandemics (SMG, 2021). Such meanings compel government agencies to intervene swiftly and collaborate on strategies to permit dine-in patterns before it is too late. Consumers spent the recommended 14 days (14) dining out after receiving second-dose vaccines from Sinovac, Pfizer, Sinopharm, and AstraZeneca, whereas 28 days elapsed after receiving single-dose vaccines from Moderna, CanSino, and Johnson and Johnson (The Straits Times, 2021). During control movement orders, vaccinated parents who already received a single or double dosage can dine in with their 17-year-old children. According to Hassan (2021), only individuals aged 18 years and older are eligible to receive the COVID-19 immunization during the present epidemic in Malaysia. Malaysia is 16th in the world in terms of fatalities, whereas India ranks as No. 1 (MALAYSIA NOW, 2021). As a result, Malaysia’s government expands the scope of the conditional movement control order to include movement control orders, which will aid in containing the spread of COVID-19.

### Scenario Building

Three elements of threat appraisal—perceived vulnerability, altruistic fear, and anticipated regret—were proposed as one strategy for achieving the present research’s objectives based on significant extant research on the COVID-19 pandemic. The COVID-19 scenario represents situations connected with psychological fear, resulting in customers shifting their preferences away from dine-in restaurants toward online delivery or takeout. The COVID-19 scenario encompasses switching intentions resulting in customers’ changing behaviors and brand awareness are critical to moderating their relationship. Using six meals, a pilot test was done with 250 Malay diners using convenience sampling to validate their fear of COVID-19. Three things were examined in order to rule out the dine-in fear scenario (i.e., “the numbers of diners at this restaurant were excessive compared to capacity”). The remaining three products were employed in the scenario of delivery and carryout (i.e., “the COVID-19 parameter of social distancing at this restaurant was overlooked, so we shifted to online delivery”). Based on the results of the pilot research, scenarios were assessed for improved comprehension and are reported in Table 1.

**TABLE 1:** Dine-in and online delivery/carryout scenarios.

Dine-in:
“You arrive at the restaurant to dine-in. When you entered the restaurant, you notice that the restaurant has been overcrowded that trigger psychological fear. They usually don’t have a dedicated safety professional because they do not change gloves, wiping each table with a fresh towel, and wearing masks. Their menus and everything that goes on the table has not to be sanitized so they avoid making eye contact with you. Instead of bothering safety measures, the staff behavior is critical with other diners. You notice their uniform is not clean and they don’t offer any apology or explanation.”
**Online delivery/carryout:**
“You see that restaurants have added delivery and carryout to their services for consumers to enjoy food from their favorite spots and such services paved a way for switching dine-in toward online delivery or carryout. Even providing carryout options also increases supply costs but restaurants continue to make changes due to COVID-19. You notice that online food delivery is another prominent alternative of dine-in so a number of popular online food delivery services like GrabFood, foodpanda, AirAsiaFood etc., played a remedial role in current pandemic scenario.”

### Survey Design

The wide-ranging studies used a two-sectioned structured questionnaire. The first component inquired for general demographic information (gender, age, education, occupation, monthly income, purchase decision, and ordering frequency) and their online patterns for dine-in and purchase during the pandemic. Explicitly, “perceived vulnerability” was assessed *via* three items adapted from Zhao et al. (2016). The “altruistic fear” was measured with four items adapted from Kurzban et al. (2015). The “anticipated regret” was evaluated with five items adapted from Heitmann et al. (2007). The “switching intention” was assessed with three items adapted from Chang et al. (2017). The “brand awareness” was measured with four items adapted from Severi and Ling (2013), and “switching behavior” was assessed with three items adapted from Malhotra and Kubowicz Malhotra (2013). There is also a section for “perceived vulnerability” and “altruistic fear.” A set of items was used to assess “perceived vulnerability,” “altruistic fear,” and “anticipated regret,” as well as “switching intention,” “brand awareness,” “switching behavior,” and “switching intention” (2013). Back-translation was used to create two instruments (one in Malay, one in English) (McGorry, 2000). The researcher translated the entire instrument from English into Malaya with the help of a language expert. Table 2 explains the measurement in detail.

**TABLE 2:** Measurement instruments.

Perceived vulnerability (Zhao et al., 2016)
PV1: Due to the detrimental reaction of COVID-19, I feel vulnerable.
PV2: I observe that I am suffering COVID-19.
PV3: I perceive that COVID-19 put harmful effect on the life of my children.
**Altruistic fear (Kurzban et al., 2015)**
AF1: I always trying to help others without any hesitation.
AF2: I feel happiness to assist others at any stage.
AF3: I will frankly help others in their difficulties.
AF4: I will intentionally assist my surrounding people.
**Anticipated regret (Heitmann et al., 2007)**
AR1: Once I purchase something then we started thinking about their alternatives.
AR2: After selecting a product, I was curious that I choose undesired one.
AR3: I feel nervous from others when they expect me that I choose better than that.
AR4: Even my choice is good but I feared that I am missing the better option.
AR5: I would escalate the offers of rivals after selecting a product.
**Switching intention (Chang et al., 2017)**
SI1: I will switch to online delivery from dine-in to fulfill my hunger needs.
SI2: I will switch from dine-in to online delivery to handle the needs of my near future.
SI3: I strongly suggest the others to do online delivery.
**Brand awareness (Severi and Ling, 2013)**
BA1: I have no issue to concepting nourishing food in my mind.
BA2: Whenever I feel hunger, I always recall only notorious food.
BA3: Only nutrient-rich food comes to my mind when I am thinking about my favorite food.
BA4: I feel pleasure when someone talk about my desired healthy food point.
**Switching behavior (Malhotra and Kubowicz Malhotra, 2013)**
SB1: I take up enough time to buy my favorite food than my previous one.
SB2: I frequently buy good food as compared to previous food.
SB3: Currently I am spending enough time and money on my desired healthy food.

### Measurements

In the current study, we used scenario-based dine-in to online delivery/carryout in the settings of the restaurant’s service that were revised from pertinent extant research (Pedrosa et al., 2020; Mehrolia et al., 2021; Zhang et al., 2021). Initially, respondents were asked whether they had experienced psychological fear of COVID-19 and were switched from dine-in toward online delivery or carryout. The respondents signposted their adverse feelings and intention of switching regarding pandemic situations to do so. Scenario dissections were steered to authenticate the suitability of the scenarios of study. At that time, the study’s item was evaluated on the scale of 7-point Likert (7 = extremely agree to 1 = extremely disagree).

### Data Collection

The COVID-19 pandemic provides an ideal experimental setting for this research. The feedback was gathered using a web-based poll in order to ascertain the link between the constructs. According to Denscombe (2006), the online survey was considered favorable because of its low cost and rapid availability of various terrestrial coverage that has been extensively used in previous studies (Akram et al., 2018; Tariq et al., 2019). A structured questionnaire was created using Google forms and distributed to respondents since it is a viable method of collecting data during lockdown because it ensures the safety of the researchers and the respondents. The current study’s target group is limited to consumers who use online meal delivery services. Data were collected using a convenience sample technique from June 8 to 28, 2021. On June 1, 2021, Malaysia implemented the FMCO (Full Movement Control Order) to restrict nationwide movement.

Nonetheless, throughout this pandemic period, the Malaysian government was permitted to operate an e-commerce business. The questionnaire was distributed *via* Facebook, WhatsApp, and Telegram to different regions of Kuala Terengganu to elicit answers from the respondents after reading the scenario. All individuals gave their consent to participate in this study.

The current study drew a sample of Kuala Terengganu (KT) residents. KT is an administrative, royal, and economic powerhouse with two renowned public institutions that attract students from all countries. KT City is the ideal venue for this study since it is representative of the dynamic Malay population and is home to international students and professionals. The respondents were randomly assigned to one of the two situations (dine-in or online delivery/carryout) and asked whether the restaurant was visited. After reviewing Scenario 1, the respondents were asked to reconsider their attitudes about the dine-in scenario regarding their psychological fear-based connections. About 739 replies were gathered from 810 disseminated surveys, with 58 respondents declining to use the online delivery/carryout in a pandemic scenario. Finally, we obtained 681 replies for additional study, representing an 84 percent response rate. According to Nulty (2008), the response rate in consumer research is between 40 and 60%. Consequently, a total of 681 pieces of feedback were collected and analyzed (327 for dine-in and 354 for online delivery and takeaway).

## Results and Interpretation

There is little difference in the respondents’ demographic features between Scenarios 1 (dine-in) and 2 (online delivery/carryout) in terms of gender, age, education, occupation, monthly income, purchasing decision, order frequency, and internet patronage. Total valid responses for Scenario 1 were 327, with males representing 57.8 percent and females contributing 42.2%. The majority of the respondents were diners, and their purchase decisions were 53.8 percent higher in the dine-in scenario than in the ordered situation (46.2 percent). Additionally, the total number of valid responses for Scenario 2 was 354, with 56.2 percent men and 43.8 percent women. The majority of the respondents were ordered regarding their purchase decision in the online order scenario (52.3 percent vs. 47.7 percent for dine-in). Table 3 contains the complete profile of the respondents.

**TABLE 3:** Respondent’s profile.

Demographics	Frequency	Percentage	Frequency	Percentage
		
	Study 1 (*n* = 327)		Study 2 (*n* = 354)	
**Gender**				
Male	189	57.8%	199	56.2%
Female	138	42.2%	155	43.8%
**Age**				
Below 18	47	14.4%	77	21.7%
19–25	98	29.9%	73	20.7%
26–40	79	24.2%	49	13.9%
41–55	70	21.4%	78	22.1%
Above 55	33	10.1%	77	21.6%
**Education**				
Diploma	99	30.3%	79	22.3%
Graduation	109	33.3%	94	26. 6%
Master	78	23.9%	89	25.1%
Others	41	12. 5%	92	26%
**Occupation**				
Government Service	97	29.6%	97	27.4%
Private Service	88	26.8%	88	24.9%
Businessman	47	14.7%	70	19.8%
House Wife	51	15.5%	51	14.3%
Students	44	13.4%	48	13.6%
Others				
**Monthly income**				
Less than 2,500 RM	89	27.3%	73	20.6%
2,501 – 5,000 RM	97	29.6%	126	35.6%
5,001 – 7,500 RM	64	19.6%	76	21.5%
7,501 RM and Above	77	23.5%	79	22.3%
**Purchase decision**				
Dine-in	176	53.8%	169	47.7%
Ordered	151	46.2%	185	52.3%
**Frequency of ordering**				
Less than 5 times	97	29.7%	93	26.3%
6–10 times	98	29.9%	91	25.7%
11–15 times	68	20.8%	82	23.2%
16 and above	64	19.6%	88	24.8%
**Mode of internet**				
Broadband	107	32.7%	117	33.1%
Mobile Phone Internet	136	41. 6%	106	29.9%
Both	84	25.7%	131	37%

### Assessment of the Measurement Model

This study used confirmatory factor analysis (CFA) to evaluate the measures’ reliability and validity. The reliability testing was assessed using the composite reliability method, while the validity assessments were performed using global fitness indexes and discriminant validity. Those approaches need to be fulfilled initially before executing the full structural equation modeling (SEM) method for hypothesis testing (Rahlin et al., 2019). This study indicates that measurement model assessment is a satisfactory range of model fit (χ^2^ = 477.212; χ^2^/df = 2.485; SRMR = 0.037; GFI = 0.919; AGFI = 0.832; NFI = 0.942; RFI = 0.938; CFI = 0.937; TLI = 0.934; RMSEA = 0.059) (Hu and Bentler, 1999). The standardized factor loadings (SFL) of all the items exceeded the threshold level of 0.50 at a significance of *p* < 0.001. The value of average variance extracted (AVE) fell within the range of 0.665–0.793 that also meets the standard value of 0.60. All values of composite reliability (CR) were fell within the range of 0.858–0.920 that also exceeded the threshold value of 0.70 (Hair et al., 2011). According to Nunnally (1978), the Cronbach’s alpha (α) coefficients must be greater than equal to 0.70, and each construct alpha ranges between 0.78 and 0.94. Thus, according to Fornell and Larcker (1981), the convergence validity was supported regarding the present study measures (see Table 4).

**TABLE 4:** Results of confirmatory factor analysis.

Construct	Indicators	SFL	AVE	CR	A
Perceived vulnerability	PV1: Due to the detrimental reaction of COVID-19, I feel vulnerable.	0.92	0.793	0.920	0.91
√AVE = 0.89	PV2: I observe that I am suffering COVID-19.	0.87			
	PV3: I perceive that COVID-19 put harmful effect on the life of my children.	0.88			
Altruistic fear	AF1: I always trying to help others without any hesitation.	0.79	0.674	0.892	0.89
√AVE = 0.82	AF2: I feel happiness to assist others at any stage.	0.87			
	AF3: I will frankly help others in their difficulties.	0.84			
	AF4: I will intentionally assist my surrounding people.	0.78			
Anticipated regret	AR1: Once I purchase something then we started thinking about their alternatives.	0.79	0.665	0.908	0.78
√AVE = 0.81	AR2: After selecting a product, I was curious that I choose undesired one.	0.74			
	AR3: I feel nervous from others when they expect me that I choose better than that.	0.89			
	AR4: Even my choice is good but I feared that I am missing the better option.	0.81			
	AR5: I would escalate the offers of rivals after selecting a product.	0.84			
Switching intentions	SI1: I will switch to online delivery from dine-in to fulfill my hunger needs.	0.89	0.748	0.899	0.79
√AVE = 0.86	SI2: I will switch from dine-in to online delivery to handle the needs of my near future.	0.79			
	SI3: I strongly suggest the others to do online delivery.	0.91			
Brand awareness	BA1: I have no issue to concepting nourishing food in my mind.	0.86	0.668	0.858	0.89
√AVE = 0.82	BA2: Whenever I feel hunger, I always recall only notorious food.	0.82			
	BA3: Only nutrient-rich food comes to my mind when I am thinking about my favorite food.	0.77			
	BA4: I feel pleasure when someone talk about my desired healthy food point.	0.81			
Switching behavior	SB1: I take up enough time to buy my favorite food than my previous one.	0.75	0.676	0.861	0.94
√AVE = 0.82	SB2: I frequently buy good food as compared to previous food.	0.82			
	SB3: Currently I am spending enough time and money on my desired healthy food.	0.89			

*√AVE, Discriminant validity; AVE, Average Variance Extracted; SFL, Standardized Factor Loadings; α, Cronbach’s alpha; CR, Composite Reliability.*

### Results of Confirmatory Factor Analysis

The correlation coefficients, mean, and standard deviation of the present research constructs are presented in Table 5. Results revealed that the correlation analysis of “perceived vulnerability, altruistic fear, and anticipated regret” was confidently associated with “switching intention.” “Switching intention” was also positively interrelated with “switching behavior.” “Brand awareness” was empathetically linked to “switching intention” and “switching behavior.” The results of Table 5 indicated that the square root of AVE values was greater than the correlation coefficients, so all the measures supported the discriminant validity (Fornell and Larcker, 1981).

**TABLE 5:** Discriminant validity.

Construct	Mean	SD	VIF	1	2	3	4	5	6
1	8.7801	0.83101	1.132	**0.89**					
2	9.1224	0.98102	1.257	0.31	**0.82**				
3	8.2211	0.91119	1.331	0.12	0.41	**0.81**			
4	8.8933	0.94195	1.111	0.34	0.37	0.40	**0.86**		
5	9.2341	0.88953	1.771	0.21	0.23	0.22	0.29	**0.82**	
6	9.8314	0.93223	1.423	0.33	0.29	0.27	0.28	0.49	**0.82**

*1, Perceived vulnerability; 2, Altruistic feat; 3, Anticipated regret; 4, Switching Intention; 5, Brand awareness; 6, Switching Behavior. The bold numbers in diagonal rows are square root of average variance extracted (AVE).*

### Discriminant Validity

#### Common Method Biases

Each variable’s typical method of variance approximation was measured *via* Herman single factor (Podsakoff and Organ, 1986; Podsakoff et al., 2003). Data were collected through a standard method, so counterfeit variance was evaluated across the constructs. All variable items’ exploratory factor analysis highlighted that two factors accumulated explained 55.73% of the variance in variables, with the primary factor rating being 34.39% and the subsequent factor explaining 21.34% of the total variance. Hence, the first-factor variance is less than 50%, which is below the threshold level. So, these data are pure and free of common method biases.

#### Structural Model

SEM through SPSS AMOS (24.0) was used. At a 95% confidence interval, a bootstrapping technique was applied to categorize the impact of perceived vulnerability, altruistic fear, and anticipated regret on switching intention and switching intention as a mediator in the association between threat appraisal facets and switching behavior. The effect of brand awareness as a moderator was also identified in the relationship between switching intention and switching behavior. The results of the hypothesis testing are presented in Table 6. Results indicated that goodness of fit indices were: [χ^2^(192) = 477.212; χ^2^/df = 2.485; SRMR = 0.037; GFI = 0.919; AGFI = 0.832; NFI = 0.942; RFI = 0.938; CFI = 0.937; TLI = 0.934; RMSEA = 0.059] (Hooper et al., 2008). Based on the large sample size, the goodness of fit indices and ChiSq/df value fall within the acceptable range of model fit. The structural model results signpost that association is capably considered among present study constructs (Hair et al., 2011). The present study model exquisitely captured the 73% of switching behavior through exogenous constructs (perceived vulnerability, altruistic fear, anticipated regret, switching intention, and brand awareness).

**TABLE 6:** Hypothesis result.

Path	Standardized estimation	*t*-statistics	*p-*value	Relationship
Perceived vulnerability → Switching intention	0.477	8.612	<0.001	Supported
Altruistic fear → Switching intention	0.367	6.980	<0.001	Supported
Anticipated regret → Switching intention	0.351	4.449	<0.001	Supported
Switching intention → Switching behavior	0.512	5.112	<0.001	Supported
Perceived vulnerability → Switching behavior	0.481	7.170	<0.001	Supported
Altruistic fear → Switching Behavior	0.365	2.228	<0.001	Supported
Anticipated regret → Switching behavior	0.176	1.793	0.073	Not supported

#### Results of Hypothesis Testing

Present research employed the variance inflation factor test to consider the multicollinearity issue, and Table 5 results revealed that it falls within the range of 1.111–1.423, which meets the threshold value of 3. Findings revealed that perceived vulnerability, altruistic fear, and anticipated regret all have a positive effect on switching intention [(coefficient = 0.477; *t* = 8.612; *p* 0.001)], [(coefficient = 0.367; *t* = 6.980; *p* 0.001)], and [(coefficient = 0.351; *t* = 4.449; *p* 0.001)], so H1, H2, and H3 were supported. H4 was also supported because switching intention positively affected the switching behavior (coefficient = 0.512; *t* = 5.112; *p* < 0.001). The direct relationships of perceived vulnerability and altruistic fear with switching behavior were also positive (coefficient = 0.481; *t* = 7.170; *p* < 0.001), (coefficient = 0.365; *t* = 2.228; *p* < 0.001), but the direct relationship of anticipated regret with switching behavior was negative (coefficient = 0.176; *t* = 1.793; *p* < 0.073). The structural equation model (SEM) was also suitable with goodness of fit indexes [χ^2^(198) = 477.217; χ^2^/df = 2.799; RMSEA = 0.058; GFI = 0.921; AGFI = 0.831; NFI = 0.942; IFI = 0.936; CFI = 0.938; TLI = 0.934; RFI = 0.938; PCFI = 0.799; PNFI = 0.789] as shown in Table 6.

**FIGURE 1:**
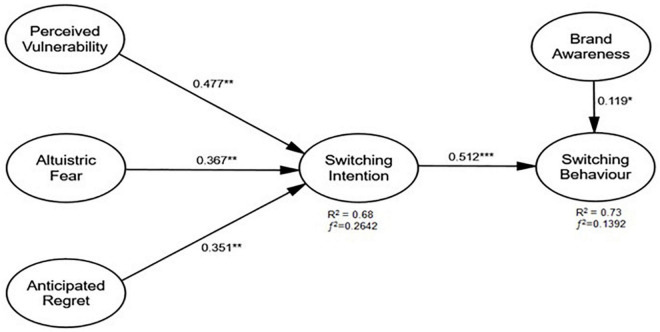
Standardized structural equation parameter estimates. *, **, *** Level of significance at 10%, 5% and 1% respectively.

Through changes in the exogenous constructs, the total variance in the endogenous construct is highlighted with the value of R2, and the results indicate a 68% change in switching intention and 73% change in switching behavior. The threshold value of R2 is 60% (Hair et al., 2011), which shows the dependent variable’s total variance. The substantial effect of the present study model was detected by examining the effect size (*f*^2^). The threshold value for small was 0.02, the medium was 0.15, and the large effect size was 0.35, as recommended by Cohen (1988) to notify the existing object among the population. The model of this study showed that switching intention (*f*^2^ = 0.2642) has a medium effect size, and switching behavior (*f*^2^ = 0.1392) has a small effect size (Figure 1).

#### Mediation Effect

The mediation analysis is performed using the bootstrap-based maximum likelihood estimator to generate the standard error of an estimate (Aimran et al., 2017; Mohamad et al., 2019). The standard error results will be eventually used for determining the critical ratio of an estimate. Using this method, results indicated a substantial impact of mediation and switching intention on the association between perceived vulnerability and switching behavior (coefficient = 0.244; *p* < 0.001). Also, switching intention on the relationship between altruistic fear and switching behavior (coefficient = 0.188; *p* < 0.001) and switching intention on the relationship between anticipated regret and switching behavior (coefficient = 0.180; *p* < 0.001) were supported because the indirect effect was found to be statistically significant, so H5 was also supported.

#### Moderation and Simple Main Effect

The metric variable moderation of the metric variables was tested *via* interaction effects using SPSS. In this procedure, we tested the direct main effects of exogenous variables (switching intention) and moderating variables (brand awareness) on the endogenous construct (switching behavior). Initially, the direct effect of switching intention on switching behavior is statistically substantial (*F* = 11.112, *p* < 0.001). The operator symbol for *p* value is (*P ≤ 0.05, **P ≤ 0.01, ***P ≤ 0.001, and ****P ≤ 0.0001). The interaction between brand awareness and switching intention (brand awareness × switching intention) statistically affects switching behavior (= 0.119, *t* = 2.112, *p* 0.05). Since the interaction effect was attained significantly, we over-tested the interaction nature for simple main effects. We tracked the suggestions of Aiken and West (1991) for this purpose. The dataset for the moderating construct was divided into groups of low and high through dummy variables. Then, we tested the intention of switching behavior at high and low levels of professional commitment. As anticipated, switching intention had a negative impact on switching behavior when brand awareness had high levels (β = −0.532, *t* = −0.341, *p* < 0.001). When employees had low levels of brand awareness, switching intention was positively related to switching behavior (= 0.532, *t* = 3.611, *p 0*.001). Furthermore, we tested the variations among both high and low levels of brand awareness. The slope variation was also substantial in the case of switching intention (*t* = 4.67; *p* < 0.001). Thus, H6 was supported.

## Discussion and Implications

The findings indicated that threat appraisals of customer valuations indicated that the level of COVID-19 psychological fear accurately characterized the consumers’ propensity to switch. It could be predicated on the time, as the data were gathered during a lockdown. Because there was a risk of contracting COVID-19 in public settings, people chose to stay at home, which resulted in online delivery. According to a recent study, perceptions of severity motivated respondents to engage in self-isolation and social withdrawal even when the vulnerability was not present (Farooq et al., 2020). As previously said, the outcomes of the study reflect the current research findings that consumers’ severity risk perceptions of COVID-19 were forced on them as a result of this life-threatening disease. COVID-19’s sternness positively affects the restaurant pattern’s switching intention, influencing consumer behavior in this study. According to Chan et al. (2020), severe COVID-19 danger enables consumers to respond to threat sensations by engaging in different behaviors such as online meal delivery or carryout rather than dining in. Thus, there is a positive relationship between threat appraisal and intention to switch.

Prior research also supports the notion that threat appraisal—altruistic fear motivated the consumers to participate in defensive behaviors such as social withdrawal and, according to Sloan et al. (2020), wearing a mask for themselves or their community in general. The findings may be unsurprising, given that customers typically seek out branded foods to satiate their cravings during pandemics. Thus, behavior toward dine-in vs. online delivery service could well be explained by the other factors associated with fear of COVID-19. The outcomes of this study corroborate previous studies utilizing protection motive theory in the COVID-19 setting, where threat appraisal is a significant predictor of protective response (Teasdale et al., 2012; Farooq et al., 2020).

These findings concur with prior research, indicating that the consumers who perceived a severe pandemic were more likely to engage in defensive behavior than those who assessed a low-risk level (Teasdale et al., 2012). Similarly, the current study found that restaurant patrons who have a psychological fear of COVID-19 are more likely to switch to online delivery services. According to Madahi and Sukati (2016), consumers’ favorable attitudes toward a product are influenced by the product’s high switchability. Thus, in both the dine-in and online food delivery/carryout situations, the mediating effect of switching intention between threat evaluation and switching behavior was positive. As a result, the current study’s findings revealed the considerable influence of switching intention on switching behavior in dine-in and online meal delivery contexts.

Additionally, the current study discovered that consumer attitudes toward online delivery in the event of a COVID-19 pandemic positively affect behavioral intention to alter dine-in practices. Since COVID-19, customers have not accepted online meal delivery as a norm. It is vital to utilize online food purchases during a lockdown that could significantly impact future food purchases. Apart from environmental concerns, Vongurai (2020) asserts that attitude is critical to consumers’ brand selections. The current data indicate that the significant relationship between intentional switching and behavioral switching is larger for online delivery than for dine-in. Brewer and Zhao (2010) assert that the primary component of brand awareness occurs when consumers see or hear about the brand’s precise identity. This way, brand awareness helps consumers’ brand selection decisions. According to Shabbir et al. (2017), brand awareness indicates that buyers emotionally connect to the product they are considering. As a result, such understanding affects consumers’ intentions to switch to online food delivery.

Finally, the findings of this study demonstrate a substantial relationship between perceived vulnerability, altruistic fear, and restaurant switching behavior. Nonetheless, no significant relationship between anticipated regret and switching behavior was discovered. Likewise, it indicated that online delivery nullifies the COVID-19 threat and gains popularity among consumers as an alternative to dine-in. To this end, self-confidence in one’s ability to switch behavior contributes to explaining the external resources required to switch behavior. As a result, online food delivery is a sensible solution to avoid COVID-19, which creates the opportunity for dine-in switching behavior.

### Theoretical Implications

The current research provides significant theoretical contributions to the literature on psychological fear and intention switching, focusing on restaurant patterns. All hypotheses were found to significantly affect the fundamental link between anticipated regret and switching behavior. Initially, this research expands the behavioral model by incorporating it into the framework of protection motive theory. Whereas previous researchers employed the idea of planned behavior in the history of consumer research, we combined the PMT framework in the context of the COVID-19 pandemic to examine the restaurant’s consumer perspective. Second, by taking into account, restaurant patrons’ fear of dining in are identified during the COVID-19 pandemic.

In contrast to previous research on restaurant settings, this study advances the field by taking the lead and analyzing the consumer’s restaurant environment from two perspectives (dine-in and online delivery/carryout). The restaurant industry’s largest segment describes how restaurant patrons migrate to dining in during a pandemic. Thus, by contrasting consumers’ dine-in and online delivery preferences, the current research laid the way for a more compassionate understanding of customers’ various inducements and attitudes toward switching behaviors during a pandemic. Thirdly, consumers’ behavior research recommends evaluating the restaurant’s facilities while keeping their thoughts and related elements of self-brand in mind (Whan Park et al., 2010). Brand awareness has yet to garner scholarly attention in restaurant contexts. Thus, the current study contributes to our understanding by examining the consumer’s response on both ends, i.e., switching intention and switching behavior. Fourth, existing research has focused chiefly on switching intention as a component of the consumer’s behavioral intention. As such, this study directs this research line, incorporates the most prevalent links involving switching intention, and examines threat evaluations’ direct and indirect relationships with switching intention and switching behavior. Finally, Malaysia ranks first in the current research location, with 82 percent of the world’s population ordering food (Jourden, 2019). Due to the cultural and restaurant environment variations between Malaysia and industrialized countries, the current study provides valuable empirical evidence on Malaysian consumer behavior. Malaysia began the restaurant industry’s rapid development phase, while it entered the rapid development phase. It rapidly extended its chain of internationally renowned restaurants, including Akar, DC restaurant, Entier French, Fuego, Gooddam, Kayuputi, Pappa Rich, Sangkaya, and LI restaurants, putting competition on a par with never-before-seen levels of ferocity (Lim, 2021). As a result, improved insights into the behavior of Malaysian and foreign consumers should be beneficial to Malaysian brands and international restaurant franchisors wishing to grow their operations.

### Managerial Implications

There are several managerial implications to discussing the difficulties confronting the restaurant business. First, diners’ psychological apprehension associated with dining-in can influence their good attitude toward and intention to place orders online. This condition implies that a pandemic might rapidly alter consumers’ dine-in behaviors and precipitate a shift away from traditional dine-in patterns toward home delivery (Ali et al., 2021). While several large and several small restaurants were forced to close due to the pandemic, the majority have successfully shifted to online services. As a result, such conversion to online delivery will continue even beyond COVID-19. Restaurants, predictably, would prioritize new internet offerings by providing superior customer experiences (Barman et al., 2021). Second, this study sheds some light on the significant motivations of restaurant consumers during the COVID-19 pandemic, such as Grab Food, Food Panda, AirAsia Food, and SmartBite. Consumers’ perceptions of switching behaviors are particularly significant when comparing vulnerability and severity concerning the COVID-19 pandemic (Youn et al., 2021). Thus, the current research advises service providers’ mechanisms for emphasizing safe and alternative scenarios for avoiding pandemics and offers various benefits to entice consumers to move online and maintain this behavior. Third, the current research has a dormant conclusion for COVID-19-related societal crusades, such as social estrangement within the community. We urge that community members make safety pleas *via* posts on the COVID-19’s seriousness and fear of altruism to spread positive attitudes toward switching behavior. Thus, their switching intentions influence consumers’ future protective behavior (Kleitman et al., 2021). By doing so, public health agencies demonstrated how effective such conduct is in combating the COVID-19 pandemic. Finally, restaurant managers working in the healthy food business must be able to adapt to consumers’ shifting behaviors and work energetically to build brand recognition by imparting knowledge about healthy food and passionately attracting people.

## Limitations and Future Research Directions

The current research has theoretical and methodological constraints that should serve as a foundation for future research. In terms of theory, the suggested model is predicated on the uniqueness of the switching intention relationship. It aided in the persistence of the current investigation, as information about the switching transition’s steering identity is rare. Nonetheless, it took a consistent stance in assessing the same alteration of future customer sentiments. Additionally, it paved the path for struggling firms to establish a presence in the market. Another theoretical constraint is that consumers’ switching intentions are unrelated to their behavior, such as alternative attraction and switching cost. Additionally, observing how such a shift in consumer behavior affected their buy intention and loyalty would be fascinating.

In survey-based investigations, cross-sectional data with common approach biases are rational. According to Samala and Singh (2019), the acceptability of a cross-sectional research survey is unaffected, a finding that is also evident in studies addressing brand challenges. While caution was exercised during the survey and in presenting the Herman single factor test score in order to mitigate the effect of common technique biases, the danger of social bias was not eradicated. As a result, we recommend that future studies collect data at multiple time intervals to forecast the precise moment and conditions that trigger a consumer behavior switch.

In terms of the sample, the researcher collected information from restaurant patrons, who are prevalent in the current pandemic context in Malaysia. In examining the COVID-19 phenomenon, the sample accurately represents a clear picture of consumer switching behavior in Malaysia. Data can also be collected from the general audience in future studies, yielding different results. Additionally, present research took switching intention as a moderator. However, in the future, potential researchers could use different demographic variables, such as age, because age would be considered an influential factor in shifting consumers’ threat perception in a COVID-19 pandemic context. Other facets that played a significant role but have not been given sufficient attention include consumer knowledge (Wijaya and Sukidjo, 2017), family decisions, and communication patterns (Budiman and Wijaya, 2016). Still, such aspects can also be considered in the framework of protection motivation theory in the future.

## Data Availability Statement

The original contributions presented in the study are included in the article/supplementary material, further inquiries can be directed to the corresponding author/s.

## Ethics Statement

This study involving human participants was reviewed and approved by the Ethics Committee of the Department of Management Sciences, Universiti Sultan Zainal Abidin (UniSZA), Terengganu Malaysia. The patients/participants provided their written informed consent to participate in the study.

## Author Contributions

HM and AR contributed in the conception of a work. AUR and AN contributed in the acquisition, analysis and interpretation of data. IS and AA contributed in drafting the work and revising it critically for important intellectual content. All authors contributed to the article and approved the submitted version.

## Conflict of Interest

The authors declare that the research was conducted in the absence of any commercial or financial relationships that could be construed as a potential conflict of interest.

## Publisher’s Note

All claims expressed in this article are solely those of the authors and do not necessarily represent those of their affiliated organizations, or those of the publisher, the editors and the reviewers. Any product that may be evaluated in this article, or claim that may be made by its manufacturer, is not guaranteed or endorsed by the publisher.
